# Comparison of smoking traditional, heat not burn and electronic cigarettes on salivary cytokine, chemokine and growth factor profile in healthy young adults–pilot study

**DOI:** 10.3389/fphys.2024.1404944

**Published:** 2024-06-10

**Authors:** Sara Zięba, Mateusz Maciejczyk, Bożena Antonowicz, Aleksandra Porydzaj, Mariusz Szuta, Giuseppe Lo Giudice, Roberto Lo Giudice, Stanisław Krokosz, Anna Zalewska

**Affiliations:** ^1^ Doctoral Studies, Medical University of Bialystok, Bialystok, Poland; ^2^ Department of Hygiene, Epidemiology, and Ergonomics, Medical University of Bialystok, Bialystok, Poland; ^3^ Department of Dental Surgery, Medical University in Bialystok, Bialystok, Poland; ^4^ Student Research Group of Department of Restorative Dentistry, Medical University of Bialystok, Bialystok, Poland; ^5^ Department of Oral Surgery, Jagiellonian University Medical College, Cracow, Poland; ^6^ Department of Biomedical and Dental Sciences and Morphofunctional Imaging, Messina University, Messina, Italy; ^7^ Department of Human Pathology of the Adult and Evolutive Age. G. Barresi, Messina University, Messina, Italy; ^8^ Independent Laboratory of Experimental Dentistry, Medical University of Bialystok, Bialystok, Poland; ^9^ Department of Restorative Dentistry, Medical University of Bialystok, Bialystok, Poland

**Keywords:** e-cigarettes, heat-not-burn products, saliva, smoking, cigarettes

## Abstract

**Objective:** Smoking is the cause of numerous oral pathologies. The aim of the study was to evaluate the effect of smoking traditional cigarettes, e-cigarettes, and heat-not-burn products on the content of salivary cytokines, chemokines, and growth factors in healthy young adults.

**Design:** Three groups of twenty-five smokers each as well as a control group matched in terms of age, gender, and oral status were enrolled in the study. In unstimulated saliva collected from study groups and participants from the control group, the concentrations of cytokines, chemokines, and growth factors were assessed by Bio-Plex^®^ Multiplex System.

**Results:** We demonstrated that smoking traditional cigarettes is responsible for increasing the level of IFN-γ compared to non-smokers and new smoking devices users in unstimulated saliva in the initial period of addiction. Furthermore, e-cigarettes and heat-not-burn products appear to have a similar mechanism of affecting the immune response system of unstimulated saliva, leading to inhibition of the local inflammatory response in the oral cavity.

**Conclusion:** Smoking traditional cigarettes as well as e-cigarettes and heat-not-burn products is responsible for changes of the local immune response in saliva. Further research is necessary to fill the gap in knowledge on the effect of new smoking devices on the oral cavity immune system.

## 1 Introduction

It has been evidenced that cigarettes contain about 400 scientifically proven carcinogens, i.e., formaldehyde, benzene or vinyl chloride; therefore, it is not surprising that cigarette smoking has been documented to be associated with an increased risk of developing cancer, as well as elevated risk of stroke, respiratory diseases, inflammation, and weakening of the body’s immune function (West, 2017; Reitsma et al., 2021; Soleimani et al., 2022). For this reason, smoking is believed to be one of the causes of premature deaths, shortening the life of an addict by up to 8 years. According to statistics, smoking kills over 8 million people annually (Carter et al., 2015; Zhu et al., 2021).

Electronic cigarettes and heat-not-burn products, considered by the public to be less harmful, were supposed to become an alternative to traditional cigarettes. Electronic cigarettes are mechanical devices that heat special solutions for inhalation, giving the user a sensation similar to ordinary smoking (DeAtley et al., 2022; Harlow et al., 2022). The liquid of e-cigarettes mainly consists of odorless carriers (propylene glycol, glycerol), nicotine, and a wide range of flavorings (Leventhal et al., 2019). It has been demonstrated that menthol flavor is one of the most popular flavors among young users. However, the effects of flavoring agents added to nicotine-containing vapors and the underlying mechanisms are largely unknown. What is more, detailed studies have confirmed also the presence of formaldehyde, acrolein and numerous heavy metals in the composition of e-cigarettes (Hahn et al., 2014; Zhao et al., 2020).

On the other hand, heat-not-burn products have been available on the consumer market for a relatively short time. Heating tobacco, rather than burning it, is intended to decrease the production of tarry substances and their supply to the body (Ratajczak et al., 2020). A detailed analysis of the composition of the heat-not-burn products’ inserts made it possible to isolate benzene, acrolein, tobacco-specific nitrosamines (Fried and Gardner, 2020). Tobacco sticks resembling traditional cigarettes may also contain flavors that make this type of addiction more attractive. Although the opinion that the new devices for supplying nicotine to the body are less harmful is becoming increasingly controversial, their use has already become extremely popular among young adults (Kreslake et al., 2021).

Research has additionally shown that the use of new devices delivering nicotine to the body may lead to the initiation of tobacco smoking among non-smokers and the relapse of smoking among former smokers (Gomajee et al., 2019; McMillen et al., 2019). But most sinisterly, many adolescents and young adults who have never smoked have started “vaping,” becoming another population that has become addicted to nicotine through these drug delivery devices (Sayed et al., 2021).

The oral cavity is the first point of contact between the toxins contained in cigarette smoke and the human body. Long-term smoking of traditional cigarettes has been proven to lead to the release of inflammatory mediators and cytokines, which is connected with the development of various oral diseases, including precancerous conditions and periodontal disease (Zhang et al., 2019). Cigarette smoke mainly affects the balance of cytokines produced by helper T cells (Zięba et al., 2024). Importantly, the concentrations of these cytokines increase with the duration of smoking. Mokeem et al. (2018) demonstrated significantly higher levels of proinflammatory IL-1β and IL-6 in the saliva of cigarette smokers. Another study showed significant differences in the expression of salivary interleukins (↓IL-10, ↓IL-5, ↑IL-2, ↑IL-4) in traditional cigarette users (Rodríguez-Rabassa et al., 2018).

Little is known about the effects of smoking electronic cigarette and heated tobacco units on the release of inflammatory mediators in saliva. Few publications report an increase in IL-6 or PGE2 concentrations in the unstimulated saliva of e-cigarette smokers compared to non-smokers (Mokeem et al., 2018; Ye et al., 2020). There are no similar studies for heat-not-burn products.

The aim of the study presented below is to evaluate and compare the concentrations of a wide panel of cytokines, chemokines, and growth factors in unstimulated saliva samples of young smokers of traditional and electronic cigarettes as well as tobacco heating systems, with a duration of addiction of one to 3 years.

## 2 Materials and methods

The study was approved by the Bioethics Committee of the Medical University of Bialystok (permission number: APK.002.175.2023). It was implemented in accordance with the Declaration of Helsinki that defines procedures in human biomedical research. Prior to qualification and collection of diagnostic material, each participant had been informed with detailed information on the purpose and methodology of the study, and gave a written consent to participate in it.

### 2.1 Subjects

The study group consisted of 75 smokers divided into 3 subgroups: TS–25 smokers of traditional cigarettes (regular cigarettes; not light/not strong); ES–25 smokers of menthol flavor electronic cigarettes; HS–25 smokers of menthol heated tobacco products. In order to qualify for the study groups, the duration of addiction of the participants could not be less than 1 year but not more than 3 years, and each subject could be a user of only one method of delivering nicotine to the body. The control group consisted of 25 non-smokers (no history of smoking traditional cigarettes/new devices delivering nicotine to the body in the past) matched in terms of age and gender to the study groups. All of the study subjects were generally healthy young adults (under 30), without inflammatory lesions in the oral cavity, with normal body weight (BMI ranging from 18.5 to 24.9), not abusing alcohol, and not taking psychoactive drugs. Participants to the study regularly attended follow-up visits to the Department of Restorative Dentistry at the Medical University of Bialystok. The number of subjects was determined according to our previous study, assuming power of the test = 0.8 (significance level alpha = 0.05) using Fisher’s formula (Jung, 2014). Within 6 months preceding our experiment, the participants from the study and control groups had not taken any medications affecting the immune response (antibiotics, steroids, antihistamines, anti-inflammatory drugs). During the study, the participants were not using fixed orthodontic appliances, and did not have Invisalign splints, removable dentures, fixed prosthetic restorations, implants, or titanium implants.

### 2.2 Saliva collection and dental examination

The study material consisted of unstimulated saliva collected from study group as well as the control group via the spitting method. The participants had been asked not to smoke or consume food or beverages other than pure water and not to perform any oral hygiene procedures at least 2 h before saliva collection. In order to minimize the effect of diurnal rhythms on saliva secretion processes, unstimulated saliva was collected between 8 a.m. and 10 a.m. In order to eliminate the subjects’s sense of restraint, saliva collection was performed in a separate room in a sitting position, with the head slightly inclined downward and minimized movements of the face and lips. Before spitting of unstimulated saliva into a plastic centrifuge tube, each participant rinsed his/her mouth three times with water at room temperature. Saliva collected within the first minute was discarded. Before centrifugation, the volume of the spat secretion was measured (with a calibrated pipette) and the rate of saliva secretion was determined by dividing the volume of saliva in the tube by the time necessary to collect it. The saliva was centrifuged for 20 min at 4°C, 10,000 × g, and then the supernatant fluid was collected, frozen at −84°C and stored until the assays were performed, but not longer than 4 months.

Dental examination was performed upon collection of the diagnostic material in order to avoid possible contamination of saliva with blood. The oral health of smokers and the control group was assessed by means of a dental mirror and a periodontal probe. The examination was conducted under electric light and included: assessment of the condition of the lips and the mucous membrane lining the tongue and cheeks; palpation of the parotid, submandibular and sublingual salivary glands; assessment of the oral hygiene index (Approximal Plaque Index, API), papillary bleeding index (PBI), measurement of periodontal pocket depth (PPD) and the DMFT index. The latter is used to determine the number of teeth with a primary or secondary carious lesion (D–decayed), teeth extracted due to caries (M–missing), and filled (F) teeth (T). The examination was conducted by one dentist (S. Z.) who had been trained beforehand, and an inter-rater examination by another dentist (A. Z.) was performed on 15 randomly selected study participants. Based on the dental examinations conducted, 10 smokers from the study groups (due to periodontal disease) and 6 subjects from the control group (poor oral hygiene, presence of numerous dental deposits) were excluded from the experiment.

### 2.3 Biochemical methods

Salivary cytokines, chemokines, and growth factors were analysed using the Bio-Plex^®^ Multiplex System according to the manufacturer’s instructions. Bio-Plex Pro Human Cytokine Assay (Bio-Rad Laboratories, Inc., Hercules, CA, United States) is a multiplex assay based on magnetic beads whose performance can be compared to a typical ELISA. The captured antibodies directed against a specific biomarker bind covalently to magnetic beads. The coupled beads then react with the sample containing the selected biomarker. A series of rinses is performed in order to remove the unbound protein, and then a biotinylated detection antibody is added to create a sandwich complex. The final complex is formed by adding streptavidin-phycoerythrin (SA-PE) conjugate. Data from the reactions are acquired using a dedicated plate reader (Bio-Plex 200) and high-speed digital signal processor.

### 2.4 Statistical analysis

The analysis of the obtained data was performed using GraphPad Prism 8.3.0. statistical software for MacOS (GraphPad Software, La Jolla, United States). Shapiro–Wilk test was used to assess normality of distribution, and the Levene’s test was used to evaluate homogeneity of variance. A one-way Kruskal–Wallis analysis of variance (ANOVA) followed by Dunn’s *post hoc* test was used to compare the quantitative variables. Multiplicity-adjusted *p*-values were also calculated. The results are presented in box plots as the median (minimum–maximum). A significance level of less than 0.05 was assumed for the statistical analyses performed.

## 3 Results

### 3.1 Clinical and stomatological findings

There were no significant differences in age, BMI, duration of addiction, unstimulated saliva flow rate, DMFT, API, PBI, and PPD between the study groups and the study groups and the control group. Clinical and stomatological characteristics were presented in Table 1.

**TABLE 1:** Clinical and dental characteristics of subjects from the study groups and the control group (BMI, body mass index; UWS, unstimulated saliva; DMFT, Decayed, Missing, Filled Teeth; API, approximal plaque index; PBI, papilla bleeding index, PPD, periodontal pocket depth; NS, not statistically significant).

	Non-smokers *n* = 25	Traditional smokers *n = 25*	E-cigarettes smokers *n = 25*	Heat-not-burn products smokers *n = 25*	*p*
Age (years)	24.7 ± 2.4	25.3 ± 3.1	23.4 ± 3.2	23.7 ± 1.9	NS
BMI (kg/m^2^)	20.6 ± 1.7	21.9 ± 1.8	21.2 ± 1.2	20.8 ± 1.9	NS
Duration of addiction (years)	—	2.1 ± 0.3	2.3 ± 0.4	2.2 ± 0.3	NS
UWS (mL/min)	0.68 ± 0.1	0.62 ± 0.1	0.65 ± 0.1	0.69 ± 0.1	NS
DMFT	17 ± 0.23	18 ± 0.32	17 ± 0.31	18 ± 0.28	NS
API	24.56 ± 0.36	21.54 ± 0.31	23.32 ± 0.27	24.89 ± 0.32	NS
PBI	0.36 ± 0.1	0.35 ± 0.12	0.34 ± 0.1	0.34 ± 0.1	NS
PPD (mm)	2.0 ± 0.5	2.0 ± 0.5	2.0 ± 0.5	2.0 ± 0.5	NS

### 3.2 Concentrations of cytokines/chemokines/growth factors in unstimulated saliva

**FIGURE 1:**
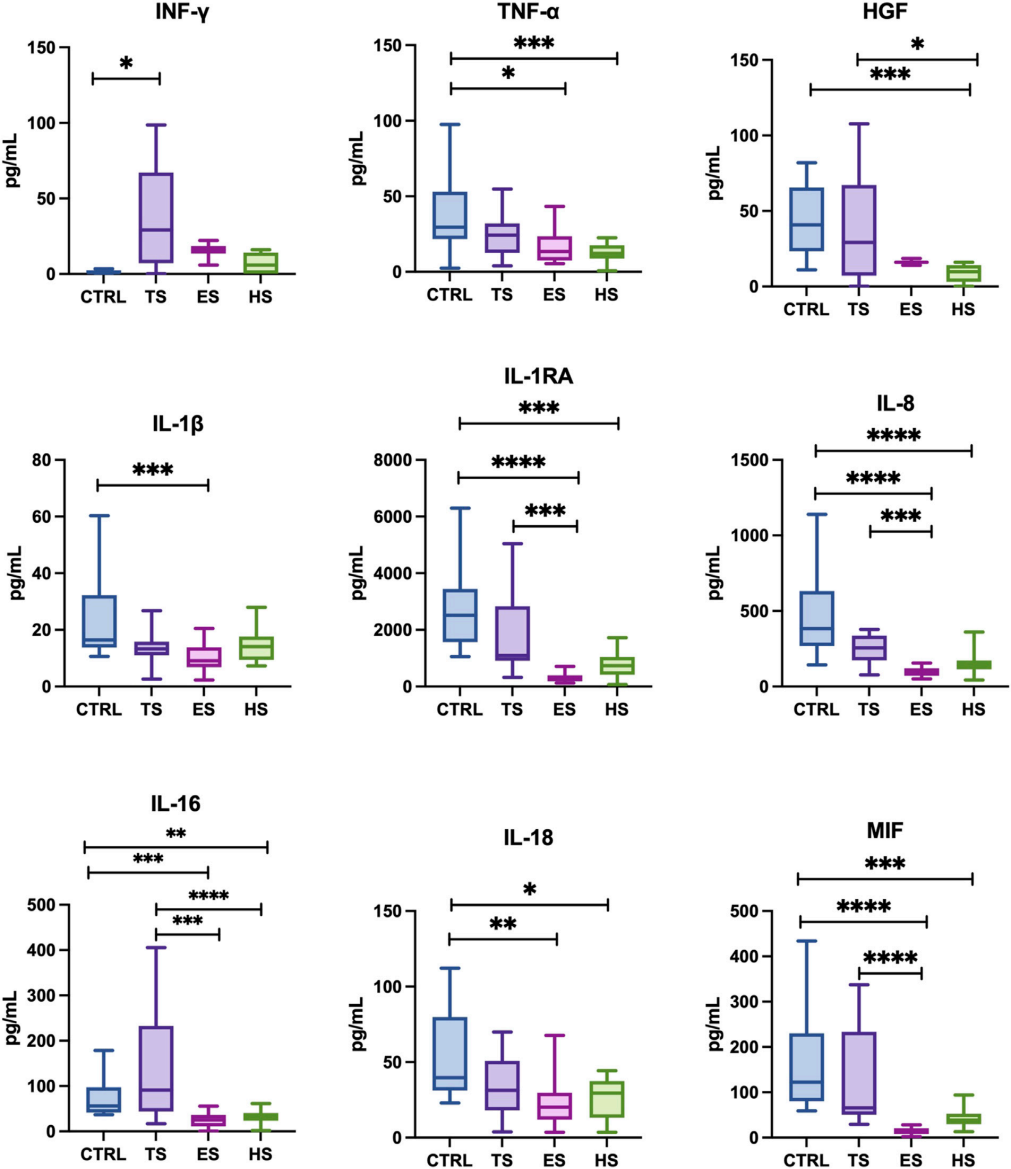
The effect of different methods of delivering nicotine to the body on the cytokine profile in unstimulated saliva: IL: 1β, 1RA, 8, 16, 18–interleukin- 1β, 1RA, 8, 16, 18; IFN-γ, interferon-γ; TNF-α, tumor necrosis factor α; HGF, hepatocyte growth factor; MIF, macrophage migration inhibitory factor; CTRL, control; TS, traditional cigarettes smokers; ES, electronic cigarettes smokers; HS, heat-not-burn products smokers. *p < 0.05, **p < 0.005, ***p < 0.0005, ****p < 0.00005.

**FIGURE 2:**
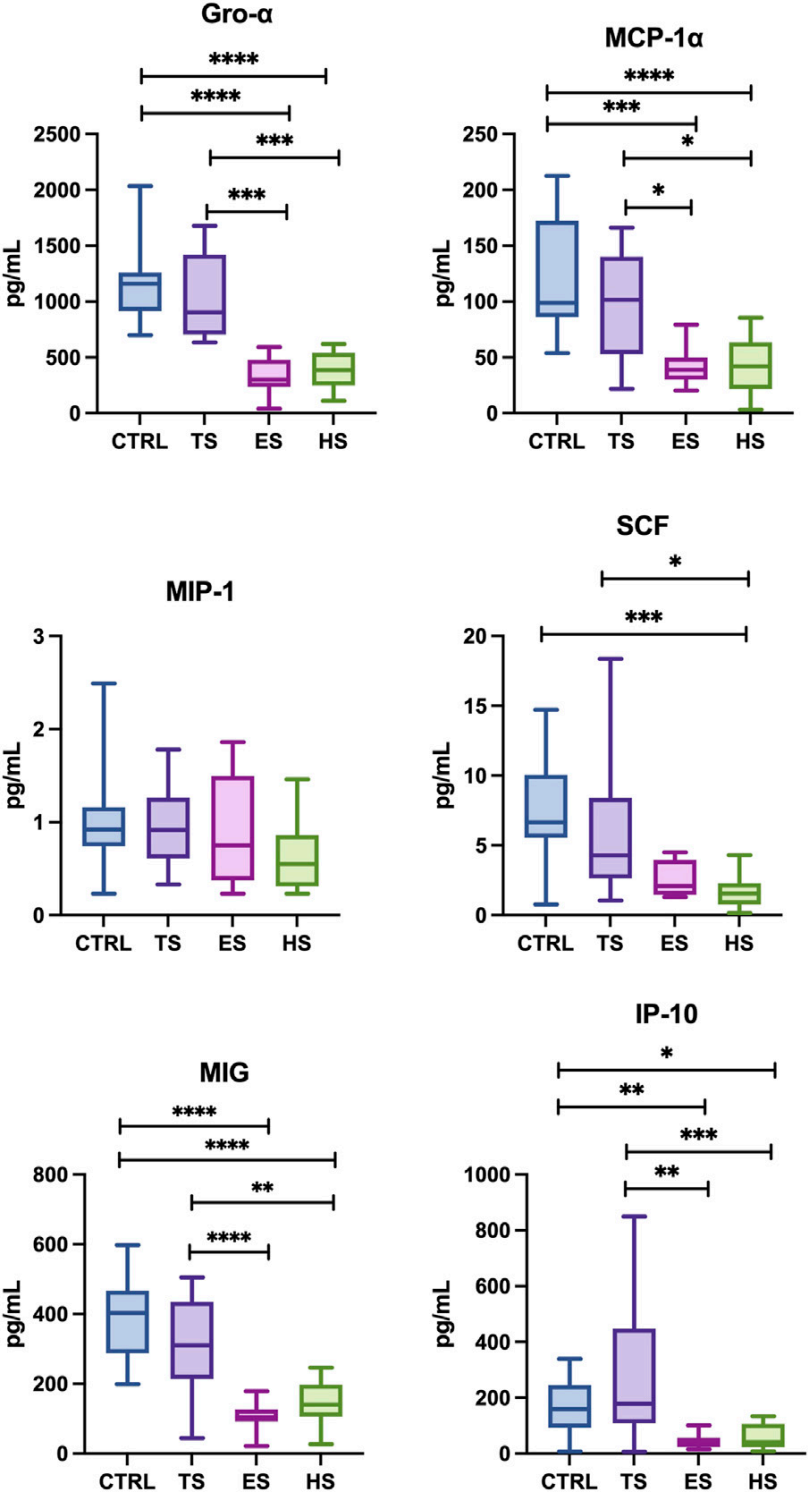
Effect of different methods of delivering nicotine to the body on the chemokine profile in unstimulated saliva: MCP-1, monocyte chemoattractant protein-1; MIP-1-α, macrophage inflammatory protein-1 alpha; GRO-α, regulated oncogene-alpha, IP-10, interferon gamma-induced protein 10; MIG, monokine induced by gamma interferon; SCF, stem cell factor; CTRL, control; TS, traditional cigarettes smokers; ES, electronic cigarettes smokers; HS, heat-not-burn products smokers. *p < 0.05, **p < 0.005, ***p < 0.0005, ****p < 0.00005.

**FIGURE 3:**
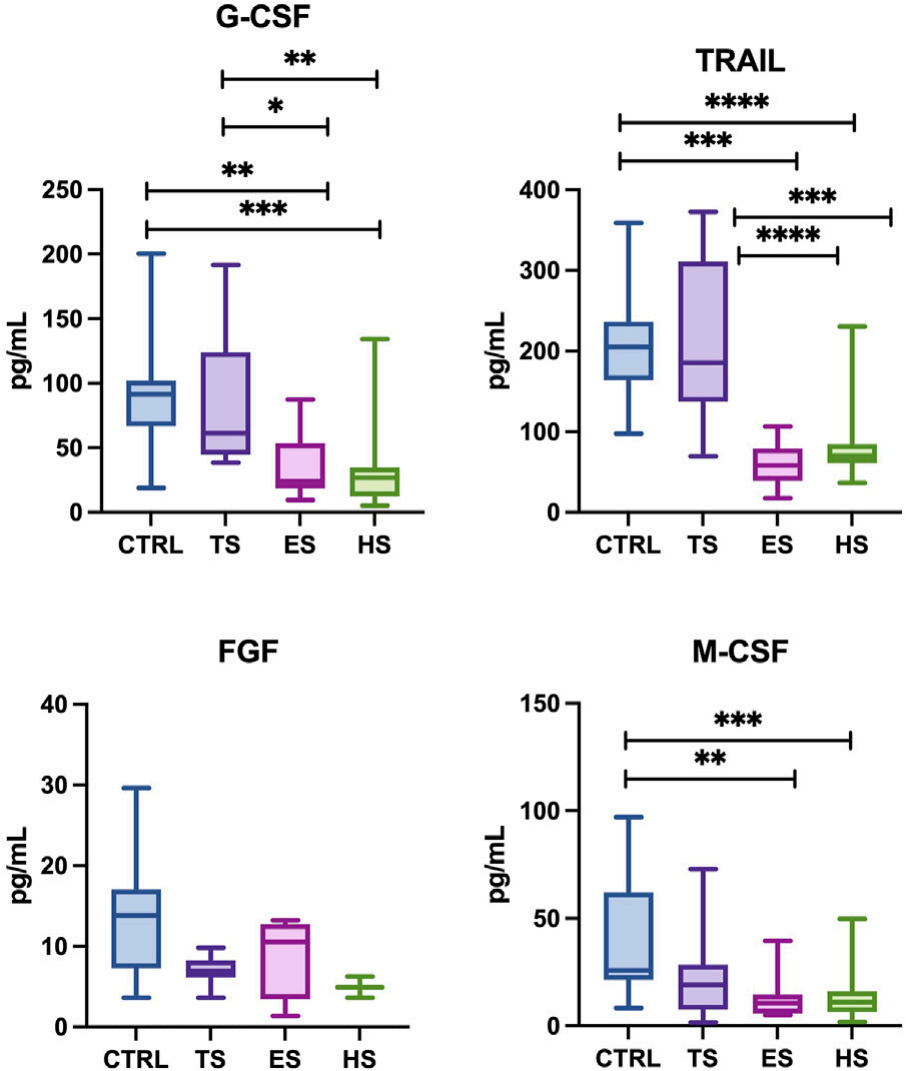
The effect of different methods of administering nicotine to the body on the profile of growth factors in unstimulated saliva: FGF, fibroblast growth factor; G-CSF, granulocyte colony-stimulating factor; M-CSF, macrophage colony-stimulating factor; TRAIL, TNF-related apoptosis-inducing ligand; CTRL, control; TS, traditional cigarettes smokers; ES, electronic cigarettes smokers; HS, heat-not-burn products smokers. *p < 0.05, ** *p* < 0.005, ***p < 0.0005, ****p < 0.00005.

The concentration of cytokines: IL-3, IL-5, IL-6, IL-12 (p40), IL-10, IL-1α, IL-2, IL-2Ra, IL-4, IL-7, IL-9, IL-12 (p70), IL-13, IL-15, IL-17, TNF-α; chemokines: CTACK, MIP-1α, B-NGF, RANTES, SCGF-B, Eotaxin, LIF, SDF-1a, and growth factors: VEGF, GM-CSF, PDGF-BB in unstimulated saliva collected from subjects was below the detection level of the assay used. Concentrations of statistically significant levels of cytokines/chemokines/growth factors in particular study groups and control group were presented in Figures 1–3 as well as in Supplementary Tables S1–S3.

#### 3.2.1 Concentrations of cytokines

Smokers of traditional cigarettes demonstrated significantly higher levels of salivary IFN-γ (↑68%, *p* = 0.0347) compared to the non-smoking controls. Other salivary cytokines (TNF-α, HGF, IL-1β, IL-1RA, IL-8, IL-16, IL-18, MIF) did not differ significantly between the two groups.

Subjects using heat-not-burn products had significantly lower content of salivary TNF-α, HGF, IL-1RA (↓57% *p* = 0.0006, ↓69% *p* = 0.0008, ↓51% *p* = 0.0001, respectively) and IL-8, IL-16, IL-18, MIF (↓54% *p* < 0.0001, ↓50% *p* = 0.0016, ↓41% *p* = 0.0222, ↓51% *p* = 0.0003, respectively.) in comparison with non-smokers. The concentration of other salivary cytokines (IFN-γ, IL-1β) between the group of heat-not-burn products smokers and the group of non-smokers revealed no statistical significance. Similary, the group of heat-not-burn products smokers was characterized by significantly lower levels of salivary cytokines HGF and IL-16 (↓54% *p* = 0.0004, ↓61% *p* = 0.0463, respectively.) compared to traditional cigarette smokers. Concentrations of other detectable salivary cytokines (IFN-γ, TNF-α, IL-1β, IL-1RA, IL-8, IL-18, MIF), presented no statistical significance between the group of tobacco-heating-system smokers and traditional smokers.

Smokers of electronic cigarettes had significantly lower levels of salivary cytokines TNF-α, IL-1β, IL-1RA (↓47% *p* = 0.0307, ↓58% *p* = 0.0008, ↓79% *p* < 0.0001, respectively.) and IL-8, IL-16, IL-18, MIF (↓78% *p* < 0.0001, ↓62% *p* = 0.0003, ↓56% *p* = 0.0019, ↓85% *p* < 0.0001, respectively.) compared to the group of non-smokers. Concentrations of the other salivary cytokines (IFN-γ, HGF) between the e-cigarette smoking group and the non-smokers did not present statistical significance. Additionally, e-cigarette users demonstrated significantly lower concentrations of salivary cytokines: IL-1RA, IL-8, IL-16 and MIF (↓72% *p* = 0.0002, ↓72% *p* = 0.0004, ↓64% *p* < 0.0001, ↓81% < 0.0001, respectively.) compared to smokers of traditional cigarettes. The levels of salivary cytokines (IFN-γ, TNF-α, HGF, IL-1β, IL-18) between the groups of e-cigarette smokers and traditional cigarette smokers showed no statistical significance.

Between the group of e-cigarette smokers and the group of heat-not-burn products smokers, no significant differences were observed in the levels of the detected salivary cytokines.

#### 3.2.2 Concentrations of chemokines

The concentrations of Gro-α, MCP-1α, SCF, MIG, IP-10 (↓68% *p* < 0.0001, ↓58% *p* < 0.0001, ↓70% *p* = 0.0004, ↓56% *p*=< 0.0001, ↓47% *p* = 0.0178, respectively.) in the saliva heat-not-burn products group were considerably lower compared to the non-smoking control group. Similary, smokers of heat-not-burn products demonstrated significantly lower levels of salivary chemokines: Gro-α, MCP-1, SCF, MIG, IP-10 (↓65% *p* = 0.0002, ↓47% *p* = 0.0253, ↓63% *p* = 0.0286, ↓48% *p* = 0.0081, ↓52% *p* = 0.0027, respectively.) compared to traditional cigarette smokers. Concentrations MIP-1α presented no statistical significance between the group of tobacco-heating-system smokers vs. non-smokers as well as tobacco-heating-system smokers and traditional smokers.

Concentrations of salivary chemokines Gro-α, MCP-1, MIG, IP-10 (↓74% *p* < 0.0001, ↓62% *p* = 0.0001, ↓73% *p* < 0.0001, ↓58% *p* = 0.0039, respectively.) were significantly lower in the group of e-cigarette smokers compared to the non-smoking controls. Additionally, e-cigarette smokers demonstrated significantly lower concentrations of Gro-α, MCP-1, MIG, IP-10 (↓51% *p* = 0.0435, ↓71% *p* = 0.0005, ↓68% *p* = 0.0002, ↓62% *p* = 0.0006, respectively.), compared to smokers of traditional cigarettes. The levels of salivary MIP-α and SCF between the groups of e-cigarette smokers and non-smokers as well as between e-cigs users and traditional cigarette smokers showed no statistical significance.

Between the group of traditional smokers vs. non-smokers as well as compared e-cigarette smokers and the group of heat-not-burn products smokers, no significant differences were observed in the levels of the detected salivary chemokines.

#### 3.2.3 Concentrations of growth factors

Heat-not burn products users had significantly lower concentrations of the salivary G-CSF, TRAIL, M-CSF (↓62% *p* < 0.0001, ↓57% *p* < 0.0001, ↓52% *p* = 0.0003, respectively) compared to the non-smoking control group. There was no statistical significance in FGF levels between those two groups. Similary, smokers of heat-not-burn products was characterized by significantly lower levels of G-CSF, TRAIL (↓57% *p* = 0.0019, ↓55% *p* = 0.0005, respectively.) compared to traditional cigarette smokers. The levels of FGF and M-CSF presented no statistical significance between the group of tobacco-heating-system smokers and traditional smokers.

The concentrations of salivary G-CSF, TRAIL, M-CSF (↓54% *p* = 0.0014, ↓71% *p* < 0.0001, ↓53% *p* = 0.0023, respectively.) were significantly lower in the group of e-cigarette smokers compared to the non-smoking controls. Concentrations of FGF did not present statistical significance. Additionally, e-cigarette smokers demonstrated significantly lower concentrations of G-CSF, TRAIL (↓48% *p* = 0.0369, ↓70% *p* < 0.0001, respectively.) compared to smokers of traditional cigarettes. The levels of salivary FGF between the groups of e-cigarette smokers and traditional cigarette smokers showed no statistical significance.

Between the group of traditional smokers vs. non-smokers as well as compared e-cigarette and heat-not-burn products smokers, no significant differences were observed in the concentractions of the salivary growth factors.

## 4 Discussion

Cigarette smoking is associated with numerous diseases and constitutes a serious challenge to the current healthcare system worldwide. Scientific sources report that up to 1/3 of the world’s population may be affected by this problem (West, 2017; Dai et al., 2022a; Dai et al., 2022b). Exposure to tobacco smoke is considered an important cause of death and is connected with the development of numerous systemic disorders, including: respiratory and gastrointestinal diseases, promotion and progression of the development of cancer, as well as local effect on the oral environment (West, 2017; Zhang et al., 2022).

The purpose of this publication was to evaluate the effects of various forms of delivering nicotine to the body on the local immune defence system of the oral cavity. It should be underlined that we still know relatively little about the influence of alternatives to smoking traditional cigarettes on our health, both in the systemic and local context, and published research results often remain contradictory.

In our experiment, the study groups consisted of both traditional cigarette smokers and users of modern methods of nicotine delivery to the body: e-cigarettes and heated tobacco products. Due to numerous modifications and marketing campaigns, the above-mentioned “new” devices that supply nicotine to the body are gaining enormous popularity among young people, leading to “renormalisation” of the smoking habit (DeAtley et al., 2022; Harlow et al., 2022; Jankowski et al., 2022). Their high popularity is influenced by the fact that there are many flavors of e-cigarette liquids/cigarette sticks for heat-not-burn products on the market (over 8,000) (Xu et al., 2022). Research shows that the menthol flavor in modern devices that deliver nicotine to the body is the most common choice among young smokers (Leventhal et al., 2019). It is worth mentioning that in most countries, regulations prohibit the sale of traditional flavored cigarettes, and similar restrictions are planned for e-cigarettes and heat-not-burn products.

For the above reasons, we qualified only young, generally healthy adults (under 30 years of age) using only one source of nicotine to participate in the study (we excluded those who can be considered mixed-smokers). Smokers of modern devices that delivered nicotine to the body used the menthol flavor. The duration of the participants’ addiction ranged from 1 to 3 years. Unstimulated saliva was used because its collection is easy, non-invasive, and quick. Its measurement does not require special equipment or expertise (Malamud, 2011; Dawes and Wong, 2019). To the best of our knowledge, this experiment is the first to evaluate the behaviour of a wide range of immune markers in the unstimulated saliva of smokers of as many as 3 (currently the most popular among young people) methods of supplying the body with nicotine. The results of our study appear all the more interesting given the widespread stereotype that e-cigarettes and heat-not-burn products are “healthier” alternatives to traditional cigarettes. The use of both alternative nicotine-delivery devices seems to clearly inhibit the local immune response in the unstimulated saliva of smokers, while smoking traditional cigarettes only slightly intensifies the inflammatory response compared to non-smokers. The results obtained should be viewed as a summary of the processes occurring at early stages of immune dysfunction in young, generally healthy individuals as a result of smoking.

The negative effects of smoking traditional cigarettes on oral health have been well documented to date. It is known that long-term smoking of traditional cigarettes affects the quantitative and qualitative composition of saliva (decreased secretion/buffering capacity, altered bacterial microflora with predominance of anaerobic bacteria, imbalance in the salivary redox status and inflammation) (Voelker et al., 2013; Animireddy et al., 2014; Zięba et al., 2022, 2024).

Cigarette smoke from traditional cigarettes induces an inflammatory response in a short period of addiction, which was manifested in our experiment by elevated concentration of INF-γ observed in traditional smokers. As indicated by the study of Rahimi et al. (2018), prolonging the time of exposure to tobacco smoke intensifies the immune system response, which indicates an increase in the concentration of subsequent proinflammatory cytokines (IL-2). This theory is confirmed by the positive correlation between the duration of smoking and the content of INF-γ in the unstimulated saliva of subjects (Rahimi et al., 2018). In light of the evidence it appears that the boost of the immune system reaction is caused by components of cigarette smoke other than nicotine. According to the results of studies, cadmium present in inhaled cigarette smoke is responsible for increased concentration of proinflammatory IL-1α or COX-2, PGE2 and IL-6 in human lung cells (Martey et al., 2004; Odewumi et al., 2015; Lim et al., 2019; Paniagua et al., 2019).

As mentioned before, both e-cigarettes and heat-not-burn products weaken the local immune response of unstimulated saliva of smokers compared to non-smokers and smokers of traditional cigarettes. Smokers using heat-not-burn tobacco systems had significantly lower levels of only some of the salivary cytokines (TNF-α, HGF, IL-1RA, IL-8, IL-16, IL-18, MIF) as well as chemokines (Gro-α, MCP-1, SCF, MIG, IP-10), and growth factors (G-CSF, TRAIL/M-CSF) assayed, compared to subjects without the smoking addition. Similarly, electronic cigarette users presented a significant decrease in the levels of salivary cytokines (TNF-α, IL-1β, IL-1RA, IL-8, IL-16, IL-18, MIF), chemokines (Gro-α, MCP-1, MIG, IP-10), and growth factors (GM-CSF, TRAIL, M-CSF) compared to those who never smoked. It is also worth mentioning that we did not observe any statistically significant differences in salivary cytokine/chemokine/growth factor levels between the group of heat-not-burn product users and e-cigarette smokers, which may suggest that their mechanism of acting on the local immune response system is similar. Moreover, the group of heat-not-burn tobacco system smokers showed lower levels of salivary cytokines (IL-16, HGF), chemokines (Gro-α, MCP-1, SCF, MIG, IP-10), and growth factors (G-CSF, TRAIL) compared to traditional cigarette smokers. In the saliva of e-cigarette smokers, we also demonstrated significantly lower concentrations of cytokines (IL-1RA, IL-8, IL-1), chemokines (MCP-1, Gro-α, MIG, IP-10), and growth factors (G-CSF, TRAIL) compared to traditional cigarette smokers. The observed inhibitory effect on the synthesis or the release of the studied cytokines from cells may be caused by the influence of the menthol additive of e-cigarettes and heat-not-burn.

Some data suggest that the menthol flavor contained in modern nicotine delivery devices may modulate cytokine expression. Xu et al. (2022) showed a strong reduction in the tested inflammatory factors in the serum of smokers of menthol-flavored e-cigarettes. Similarly to our study, the concentration of M-CSF and C5 in serum was significantly reduced in the group smoking e-cigarettes containing a mixture of nicotine and menthol compared to the control group receiving carrier and the group smoking carrier + nicotine (Xu et al., 2022).

The study performed by Sayed et al. (2021) also showed lower levels of IL-1 receptor antagonist (IL-1Ra) in saliva and Gro-α in the sputum. Reduced IL-1Ra levels in saliva may indicate an early stage of gingivitis. Because IL-1Ra has an inhibitory effect on IL-1, reduced levels may play a role in the subsequent progression of pulpitis and periodontitis (Rawlinson et al., 2000; Lu et al., 2002). On the other hand–GRO-α, a member of the CXC chemokine family, is responsible for induces neutrophil chemotaxis (Meyer-Hoffert et al., 2003). Reduction of GRO-α levels in sputum may suggest increased susceptibility to respiratory infections due to the immunocompromised state.

Supplementary to data concerning changes in inflammatory cell levels, there is a discovery indicating that the use of e-cigarettes leads to alterations in the oral microbiome, affecting both microorganisms and host cells (Chopyk et al., 2021). Studies demonstrate that e-cigarette use has an adverse impact on oral health. It is observed that circulating monocytes in e-cigarette users exhibit phenotypic changes indicative of inflammation in response to reduced e-cigarette use (Sayed et al., 2021). Following stimulation with bacterial lipopolysaccharide, decreased release of IL-8 and IL-6 has been observed, suggesting a limited capacity for an effective response to bacterial infection (Sayed et al., 2021).

The cytotoxicity induced by flavoring agents used in e-cigarettes has also been assessed on cell lines and in humans. It has been found that repeated exposure to menthol significantly reduces cell viability (Rickard et al., 2021). Therefore, further research is necessary to understand the mechanisms of toxicity of flavoring agents and chemical combinations present in modern nicotine-delivering devices.

Perhaps not insignificant is the concentration of nicotine, which is considerably higher in e-cigarettes and heat-not-burn products than in traditional cigarettes (Morean et al., 2016; Romberg et al., 2019).

Nicotine is a low-lipid molecular protein that affects cell function via nicotinic acetylcholine receptors (nAChRs). Nicotine has been shown to bind to nAChR, thus reducing the expression of TNF-α through an α7 nAChR/MyD88/NF-κB pathway in HBE16 human epithelial cell line (Churg et al., 2002; Demirjian et al., 2006). The nAChR receptor has been proven to regulate the immune response primarily via nerve X, which is referred to as the “cholinergic anti-inflammatory pathway.” Nerve X releases acetylcholine which is a cholinergic agonist of the afore-mentioned receptor (Shytle et al., 2004; de Jonge and Ulloa, 2007). However, as shown in numerous studies, nicotine is much more potent in reducing proinflammatory factors and inflammatory signals (Demirjian et al., 2006; Li et al., 2011). Naturally, the concentration of nicotine must be high enough to displace acetylcholine from its receptor. Nicotine activates the α7 receptor of the nAChR subunit, thereby inhibiting the expression of inducible nitric oxide synthase and nitric oxide via the mitogen-activated protein kinase (MAPK)/NF-κB signalling pathway (Carlisle et al., 2007). Moreover, nicotine has been shown to reduce IL-8, IL-1, and PGE 2 from human epithelial cells after stimulating the α7 subunit of nAChR. On the other hand, according to the available studies, nicotine stimulates neutrophils to produce IL-8, and induces endothelial cells to produce ICAM-1 and VCAM-1, both via nAChR activation, which, according to the authors, requires further studies (Sugano et al., 1998; Dowling et al., 2007; Li et al., 2011).

The study by Mokeem et al. (2018) suggests less harmful effects of smoking electronic cigarettes. In this experiment, the concentrations of the proinflammatory cytokines IL-1β and IL-6 in the whole saliva of smokers of traditional cigarettes were significantly higher than the levels obtained from EC (electronic cigarette) users and non-smokers. Interestingly, the levels of the tested interleukins in EC smokers reached similar values to those of non-smokers. On the other hand, Ye et al. (2020) found elevated levels of the inflammatory marker PGE2 (prostaglandin 2) in traditional smokers compared to EC users and the non-smoking group. In contrast to the above-mentioned authors, Faridoun (2019); Singh et al. (2019) showed a significant increase in IL-1β in e-cigarette smokers. However, it should be mentioned that in the said works the duration of addiction was longer, the average age of the study participants was higher, and the presence of periodontal disease was taken into account, which is not without an effect on the local immune response system.

Although in our research we did not observe significant differences in dental characteristics (Table 1) due to the young age of the participants and the pilot nature of the study, it should be emphasized that changes in the local immune system may manifest clinically in the future in people with longer history of addiction. Smoking is considered the most important determinant increasing the risk of periodontal disease (by as much as 85%), it is also responsible for the disruption of the oral immune system (Leite et al., 2018). People with a long history of smoking are characterized by deeper probing depth as well as greater loss of connective tissue attachment, bone resorption and tooth loss than non-smokers (Johnson and Hill, 2004). The diverse effects of cigarette smoking on host-pathogen interactions in the oral cavity lead to a decrease in cell-mediated and humoral immune responses, promotion of infection with microbial pathogens, they also interfere with antimicrobial therapies, and strengthen antimicrobial resistance (Sopori, 2002). It is postulated that “proper” cytokine production results in protective immunity, while “improper” cytokine production leads to tissue destruction and progression of periodontal disease (Gemmell and Seymour, 2004).

Lower concentrations of Il- 16 in gingival crevicular fluid were found in smokers with periodontal disease compared to the healthy control, and this cytokine correlates with disease severity in smokers (Tsai et al., 2005). Reduced production of the pro-inflammatory cytokine IL-18, in turn, can lead to uncontrolled bacterial and viral infections of the oral cavity (Orozco et al., 2007). In addition, being a smoker significantly affects the failure rate of implant treatment, the risk of postoperative infection and marginal bone loss as well as chronic irritation of the mucosa by components of tobacco smoke (which can lead to oral ulceration) (Zuabi et al., 1999; Naseri et al., 2020; Alade et al., 2022). The above may explain by reduced concentration of HGF in the saliva of smokers compared to the control group which we demonstrated in our research. It is well known that HGF and its receptor, MET, play a key role in promoting tissue repair, supporting osteointegration of implants and also inhibiting inflammation by improving migration and proliferation of mesenchymal stem cells (Wang et al., 2017). On the other hand, TNF-α which is a pro-inflammatory cytokine, serves as a mediator of the immune response, helping to eliminate cancer cells. Reduced levels of it may be responsible in the future, for developing precancerous lesions or oral cancers (Li et al., 2011; Singh et al., 2014).

Our research has identified several limitations. Firstly, the small size of our participant group necessitates that this study be viewed as pilot study. We ensured that participants in both the study and control groups were matched for not having systemic diseases and other pertinent factors influencing, which resulted in a smaller sample size.

Additionally, the relatively young age and short smoking histories (up to 3 years) of our participants limit the generalizability of our findings to older or long-term smokers who may experience distinct oral health ramifications. Consequently, our results might not encompass the entire spectrum of smoking-related oral health effects, especially among older individuals with prolonged smoking durations.

It is important to note that we did not differentiate between heavy and light smokers in this investigation.

## 5 Conclusion

1. Modern nicotine delivery devices, namely, e-cigarettes and heat-not-burn products, inhibit the local inflammatory response in the oral cavity of young adults with a smoking addiction lasting no longer than 3 years.
2. Both e-cigarettes and heat-not-burn products appear to act via a similar mechanism on the immune response system of unstimulated saliva.
3. Smoking traditional cigarettes slightly induces the local salivary immune response of young adults with a smoking addiction lasting up to 3 years.
4. Disruption of local immune response in the oral cavity, developing from smoking traditional cigarettes, e-cigarettes, or heat-not-burn products, may have a negative effect on smokers’ oral health in the future.

## Data Availability

The original contributions presented in the study are included in the article/Supplementary Material, further inquiries can be directed to the corresponding author.
