# Supplementary material for: Comparison of smoking traditional, heat not burn and electronic cigarettes on salivary cytokine, chemokine and growth factor profile in healthy young adults–pilot study
Source: Front Physiol. 2024 Jun 10;15:1404944. doi: 10.3389/fphys.2024.1404944 (PMC11194668; doi:10.3389/fphys.2024.1404944)
Supplement: Supplementary file 1 [file Table1.docx]

**Table S1. The effect of different methods of delivering nicotine to the body on the cytokine profile in unstimulated saliva: IL: 1β, 1RA, 8, 16, 18** – interleukin- 1β, 1RA, 8, 16, 18; **IFN-γ** – interferon-γ; **TNF-α** – tumor necrosis factor α; **HGF** – hepatocyte growth factor; **MIF** – macrophage migration inhibitory factor.

|  | **Non-smokers** | | **Traditional smokers** | | **E-cigarettes smokers** | | **Heat-not-burn products smokers** | | P value |
| --- | --- | --- | --- | --- | --- | --- | --- | --- | --- |
|  | Mean ± SD | Median  (min–max) | Mean ± SD | Median  (min–max) | Mean ± SD | Median  (min–max) | Mean ± SD | Median  (min–max) |  |
| **IFN-γ** | 1.148±0.8661 | 0.845 (0.48–2.42) | 40.04±35.34 | 29.19  (0.24–107.7) | 16.89±1.415 | 16.07  (16.07– 18.52) | 7.432±6.452 | 5.92  (0.24–16.07) | 0.0088 |
| **TNF-α** | 39.11± 25.08 | 29.51  (2.44– 97.51) | 23.79±13.54 | 24.35  (3.99– 54.85) | 16.89±12.49 | 13.48  (5.46–43.26) | 12.7  5.621 | 12.2  (0.7–22.59) | 0.0006 |
| **HGF** | 50.04±38.85 | 40.84  (11.09–176.4) | 40.04±35.34 | 29.19  (0.24–107.7) | 16.22±2.229 | 16.07  (14.07–18.52) | 8.638±5.933 | 9.82  (0.24–16.07) | 0.0015 |
| **IL-1β** | 23.33±14.62 | 16.47  (10.66–60.3) | 13.84±5.61 | 13.32  (2.62–26.78) | 10.15±5.03 | 9.07  (2.33–20.48) | 14.69±5.598 | 14.1  (7.31–27.99) | 0.0019 |
| **IL-1RA** | 2702±1423 | 2517  (1057–6299) | 1818±1535 | 1094  (321–5044) | 332.5±172.6 | 306.6  (126.3–710.4) | 768.5±427.8 | 733.9  (69.19-7234) | <0.0001 |
| **IL-8** | 442.1±244.6 | 382.7  (142.7–1139) | 245.5±90.44 | 256  (76.48–378.2) | 94.83±30.18 | 91.28  (50.42–155.4) | 152.6±66.16 | 140.4  (43.22–360.2) | <0.0001 |
| **IL-16** | 71.53±38.6 | 56.09  (37.04–178.6) | 141.6±129.8 | 90.88  (16.77–405.7) | 25.46±16.97 | 25.38  (0.59–55.68) | 31.23±14.18 | 30.28  (1.68–61.62) | <0.0001 |
| **IL-18** | 77.23±107.2 | 39.72  (22.95–479.3) | 36.12±20.01 | 31.32  (3.78–69.89) | 23.13±16.72 | 20.19  (3.54–67.7) | 26.73±12.96 | 29.43  (3.54–44.34) | 0.0016 |
| **MIF** | 192.9±182.7 | 122.1  (59.06–678.9) | 121.4±111.8 | 65.49  (29.32–337.3) | 14.77±8.437 | 14.98  (2.16–28) | 43.04±21.26 | 38.5  (13.15–93.79) | <0.0001 |

**Table S2. Effect of different methods of delivering nicotine to the body on the chemokine profile in unstimulated saliva**: **MCP-1** - monocyte chemoattractant protein-1, **MIP-1-α** –macrophage inflammatory protein-1 alpha, **GRO-α** –-regulated oncogene-alpha, **IP-10** –interferon gamma-induced protein 10, **MIG** –monokine induced by gamma interferon, **SCF** – stem cell factor.

|  | **Non-smokers** | | **Traditional smokers** | | **E-cigarettes smokers** | | **Heat-not-burn products smokers** | | P value |
| --- | --- | --- | --- | --- | --- | --- | --- | --- | --- |
|  | Mean ± SD | Median  (min-max) | Mean ± SD | Median  (min-max) | Mean ± SD | Median  (min-max) | Mean ± SD | Median  (min-max) |  |
| **Gro-α** | 1134±322.6 | 1159  (698.8–2035) | 1012±337.1 | 904.1  (634–1678) | 338.8±167.1 | 300.4  (40.5–593.6) | 392.8±163 | 387.4  (111.5–619.7) | <0.0001 |
| **MCP-1** | 124.6±51.02 | 98.92  (53.8– 212.5) | 95.07±49.51 | 101.5  (21.84– 166.1) | 42±16.48 | 38.92  (20.39– 79.33) | 43.9±24.62 | 41.91  (3.21– 85.6) | <0.0001 |
| **MIP-1α** | 1.026±0.5303 | 0.92  (0.23–2.49) | 0.9771±0.4265 | 0.915  (0.33–1.78) | 0.8922±0.6027 | 0.75  (0.23–1.86) | 0.6315±0.3766 | 0.55  (0.23–1.46) | 0.0741 |
| **SCF** | 7.559±3.754 | 6.655  (0.77–14.73) | 5.946±4.677 | 4.295  (1.05–18.37) | 2.5±1.399 | 2.09  (1.31–4.51) | 1.664±1.249 | 1.56  (0.15–4.3) | 0.0005 |
| **MIG** | 381.3±118.6 | 402.9  (199–597.9) | 314.9±134.8 | 310.3  (44.51–504.9) | 103.5±38.69 | 103.7  (21.82–179.2) | 144.1±62.03 | 140.3  (26.94–246.5) | <0.0001 |
| **IP-10** | 167.6±99.02 | 159.2  (6.85– 339.5) | 288.7±265.5 | 178.7  (6.28– 849.2) | 47.63±26.18 | 45.04  (15.13– 101.2) | 63.48±43.21 | 49.47  (10.31–134.1) | <0.0001 |

**Table S3.** **The effect of different methods of administering nicotine to the body on the profile of growth factors in unstimulated saliva**: **FGF** – fibroblast growth factor, **G-CSF** – granulocyte colony-stimulating factor, **M-CSF** – macrophage colony-stimulating factor, **TRAIL -** TNF-related apoptosis-inducing ligand.

|  | **Non-smokers** | | **Traditional smokers** | | **E-cigarettes smokers** | | **Heat-not-burn products smokers** | | P value |
| --- | --- | --- | --- | --- | --- | --- | --- | --- | --- |
|  | Mean ± SD | Median  (min-max) | Mean ± SD | Median  (min-max) | Mean ± SD | Median  (min-max) | Mean ± SD | Median  (min-max) |  |
| **G-CSF** | 100.1±60.7 | 91.62  (18.87–271.3) | 95.18±75.3 | 61.38  (38.48–266.3) | 35.49±26.14 | 23.98  (9.45–87.43) | 30.42±28.74 | 26.84  (5.05–134.3) | <0.0001 |
| **TRAIL** | 204.5±62.06 | 205.2  (97.54–358.8) | 209.6±96.73 | 185.6  (69.36–372.4) | 58.14±25.42 | 58.29  (17.36–106.5) | 80.41±40.69 | 70.33  (36.4–230.3) | <0.0001 |
| **FGF** | 13.64±7.538 | 13.82  (3.61–29.6) | 7.013±1.575 | 6,94  (3.61–9.83) | 8.918±5.239 | 10.56  (1.34–13.22) | 4.925±1.86 | 4.925  (3.61–6.24) | 0.0221 |
| **M-CSF** | 36.88±2.,98 | 25.81  (8.33– 97.06) | 21.44±18.84 | 19  (1.51– 72.95) | 12.85±9.846 | 10.54  (5.09–39.6) | 12.63±10.51 | 11.01  (1.78–49.85) | 0.0002 |
